# New strategies for prevention of HIV among Japanese men who have sex with men: a mathematical model

**DOI:** 10.1038/s41598-020-75182-7

**Published:** 2020-10-23

**Authors:** Stuart Gilmour, Liping Peng, Jinghua Li, Shinichi Oka, Junko Tanuma

**Affiliations:** 1 Graduate School of Public Health, St. Luke’s International University, Omura Susumu & Mieko Memorial, St. Luke’S Center for Clinical Academia, 5th Floor, 3-6-2 Tsukiji, Chuo-ku, Tokyo, 104-0045 Japan; 2 School of Public Health, Sun Yat Sen University, Guangzhou, China; 3 AIDS Clinical Center, National Center for Global Health and Medicine, Tokyo, Japan

**Keywords:** Epidemiology, HIV infections

## Abstract

HIV prevalence in Japan continues to increase among men who have sex with men (MSM). We built a mathematical model to describe the HIV epidemic, including acute infection and pre-exposure prophylaxis (PrEP), and projected the model to 2050. We compared current testing and treatment policies, a scenario where Japan achieves UNAIDS 90-90-90 targets, three PrEP scenarios defined by different coverage levels of 25%, 50%, and 75% among the 20% of MSM with the highest risk behavior, and combinations of these scenarios. With no change in interventions prevalence of HIV among MSM will rise to 9.0% in 2050. If Japan achieves full UNAIDS 90-90-90 targets, 84.9% of these infections would be averted. Under 50% PrEP with no expansion of testing and treatment, prevalence will reach 2.6% and 62.1% of infections would be averted by 2050. If in addition UNAIDS 90-90-90 goals are achieved, 92.7% of HIV infections would be prevented by 2050. All interventions tested in this model were cost-saving relative to the base case. Both PrEP and enhanced ART strategies can be cost-saving, and if Japan enhances its testing program for MSM and introduces PrEP, it has the potential to effectively eliminate new infections in the next 30 years.

## Introduction

HIV is a significant public health issue among men who have sex with men (MSM) in Japan. There are more than 1000 new infections per year^1^, and prevalence among MSM is estimated at between 1 and 3%^2,3^. Although the HIV epidemic among MSM started later in Japan than in other high-income countries^4^ prevalence has increased rapidly, driven by low levels of safer sexual activity^5,6^ and relatively poor access to sexual health services among MSM^7^, lack of attention to health issues among sexual minorities^8^, and social marginalization of the affected groups^9^. Stigma and discrimination persist both towards MSM^10^ and in public attitudes towards sexually transmitted cases of HIV^9^, and people living with HIV^11^. Although specific discriminatory policies do not exist in law in Japan, low levels of connection with MSM communities have been associated with lower levels of regular HIV testing^7^, suggesting a role for stigma and discrimination in preventing access to needed healthcare even where that care is free and anonymous. Previous mathematical models have predicted that HIV will reach prevalence above 10% if policy does not change^12^, although recent enhancements in policy may have seen improvements in progress towards UNAIDS 90-90-90 targets^13^. These policy changes include revisions of prevention guidelines that encourage a more MSM-focused strategy built around collaboration with NGOs^14^, revisions of guidelines for local government for managing HIV prevention activities^15^, and improved access to testing and treatment^16^. Despite this possible recent slowing of growth of the epidemic, the number of new cases remains high and high risk sexual behavior continues to be common among MSM^7^.

HIV prevention policy in Japan lags behind that of other high-income nations. Although Japan has a well-respected and highly equitable universal health coverage (UHC) system^17^, funding for treatment as prevention and other biomedical interventions against HIV remains limited^18^. Pre-exposure prophylaxis (PrEP) is not subsidized under the national health insurance system, and PrEP uptake is limited to off-label use with scarce access as a consequence. Unlike countries such as Australia and the UK, Japan lacks a comprehensive network of publicly-funded and politically supported health services dedicated to MSM, and dissemination and implementation of preventive activities remains fragmented and uncoordinated. In the absence of such innovative strategies Japan remains vulnerable to continued increases in the prevalence of HIV, and although treatment for HIV is supported financially by the government the continued increase in prevalence of HIV is likely to present a continued and growing public burden unless new and aggressive measures are implemented to control the epidemic.

This study uses a mathematical model to explore the effect of several different policy improvements on the HIV epidemic among MSM in Japan. The study estimates the future prevalence of HIV if policy continues unchanged from its current settings, and compares this future prevalence with the prevalence under a range of alternative strategies based on enhancing testing and treatment to UNAIDS recommended levels, introducing pre-exposure prophylaxis, or combinations of both. Based on estimates of the epidemiological and economic impact of these models, the study makes recommendations for Japanese policy-makers to improve Japan’s HIV response.

## Methods

**Figure 1:**
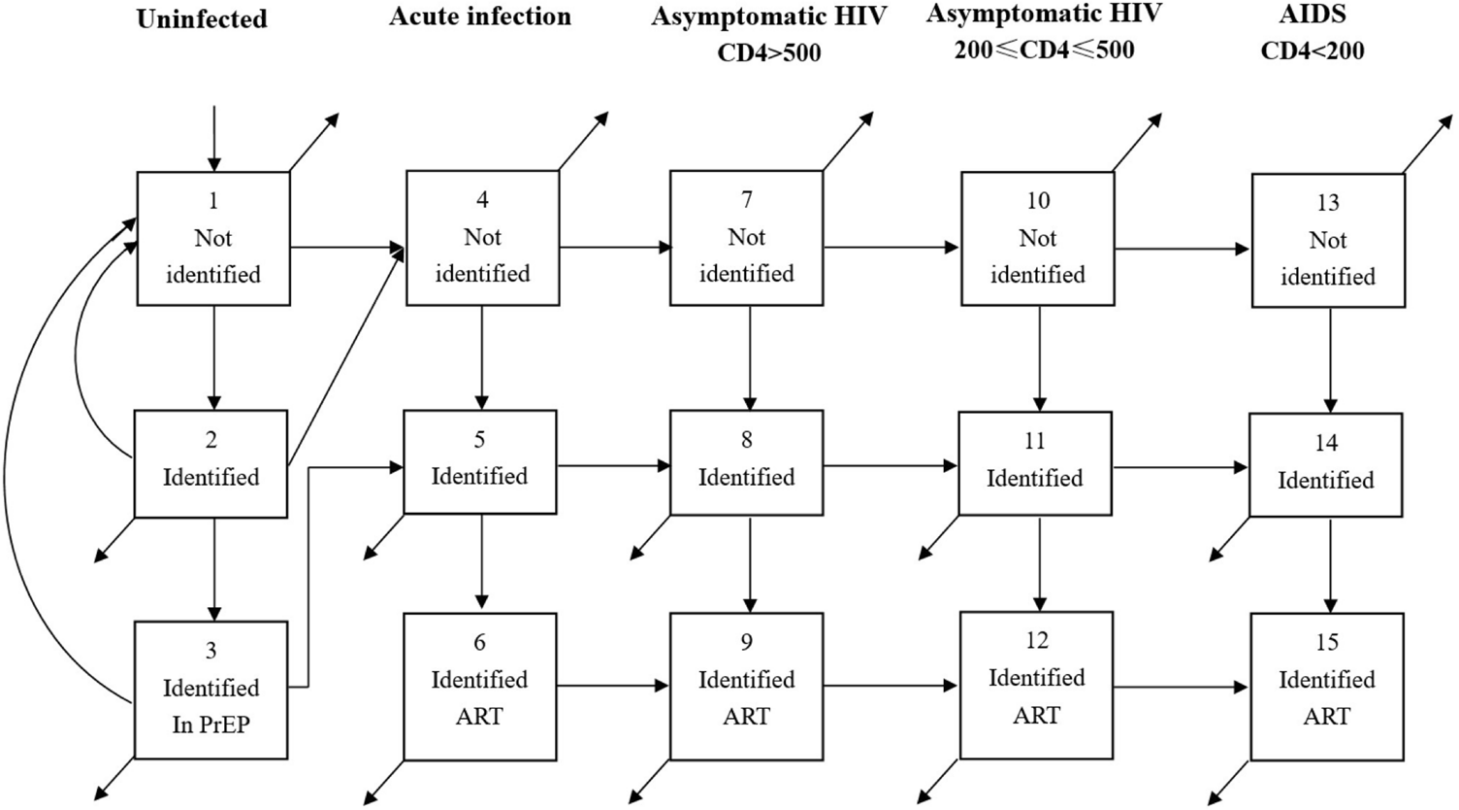
Compartmental structure of the model. Arrows indicate flow of population from one box to another. Arrows leading from a box that do not terminate in another box represent mortality/maturation.

This study used a deterministic compartmental mathematical model to describe the epidemiology of HIV in Japan. The compartmental structure divided the population of MSM into three strata of behavior based on whether they had been tested for HIV in the past year and whether they were in treatment if they had been tested positive. Every person in the model must be simultaneously in one of the three strata of HIV knowledge and one of the four stages of disease, so that for example a separate compartment exists to describe individuals who are experiencing acute HIV (stage 1 of the HIV stages) and have not identified their status (stratum 1, untested in the past year). Thus there are 15 compartments, corresponding to the four stages of HIV plus HIV-negative status, combined with one of the three strata (untested, tested and not in treatment, or in treatment). A diagram of the compartmental structure is shown in Fig. 1, with the testing strata defining the rows and the illness stages defining the columns of the compartmental structure. The four stages of HIV in people living with HIV are divided as follows:

- an acute stage which was highly infectious but short-lived
- a slow asymptomatic stage in which HIV was primarily detected by voluntary testing and counselling (VCT) that is not explicitly funded under the current national guidelines;
- an asymptomatic stage in which the disease is also detected by passive case-finding and people identified with the disease immediately enter treatment under national treatment guidelines;
- an AIDS stage in which the disease is rapidly identified and immediately enters treatment

The model was also designed to allow HIV negative individuals to be tested, and included an intervention stage for HIV-negative individuals to allow for the implementation of PrEP.

Based on the compartmental structure a system of 15 ordinary differential equations (ODEs) was defined, with each equation describing the complete inward and outward flow of population from a compartment in the model. Each arrow in Fig. 1 is described by a single term in a single equation. This model has been described in detail elsewhere^19^, and has been adapted to reflect the current state of the HIV epidemic in Japan.

The equations describing the flow of populations into and out of the compartments in Fig. 1 are described in a previous paper, which also describes the model and sensitivity analysis methods in detail^19^. Each term in each equation of this model depends on specific parameters describing testing rates, numbers of sexual partners, condom use rates, treatment entry rates and disease progression rates. These parameters were defined according to a hierarchy of data quality:

- Parameters describing disease progression and infectiousness of individual sexual contact were extracted based on data from a clinical cohort of PLWH at the AIDS Clinical Center, the National Center of Global Health Medicine in Tokyo
- Risk behavior parameters were obtained from studies of MSM at venues and in clinics in Japan
- Parameters describing testing behavior were calculated from publicly available information on numbers of tests provided by public health facilities in Japan
- Where not available from these sources, data was obtained from a search of published research on MSM both in Japan and overseas

Clinical data were collected from July 1995 to March 2017, as part of an ongoing clinical cohort collected at the National Centre for Global Health Medicine (NCGHM). Patients gave consent for their data to be collected as part of routine clinical service when first attending the clinic. Use of their data for this study received ethical approval from the NCGHM institutional review board (National Centre for Global Health and Medicine IRB approval numbers NCGM-G-002224-00 and NCGM-G-002233-00), which oversees data collection from the clinical cohorts used in this study. Study subjects gave informed consent for their clinical records to be used in research when initially enrolled in the clinical cohort. All data used in this study were anonymized, and all methods carried out in accordance with relevant guidelines and regulations.

The population was divided into a low-risk group representing 80% of all MSM and a high-risk group representing 20% of all MSM. This high-risk group was defined based on number of partners in the past year based on data from surveys and clinical cohorts in Japan. A mixing parameter was defined which enabled the model to incorporate sexual interactions between low-risk MSM and high-risk MSM^20^. All parameters used in the model and their sources are described in Table 1.

**Table 1:** Population estimates, parameter values and cost data used in the model.

Parameter	Value	References
**Demographic**		
*Initial population (aged 18–59)*		
Men	32,689,000	^30^
MSM (%)	3.4%	^31–34^
Low-risk MSM (%)	80%	^5^
High-risk MSM (%)	20%	^5^
Initial number of HIV positive MSM in 2016	14,570	^1^
Initial HIV prevalence among MSM in 2016	0.011	Calculated
*Background maturation, entry and mortality rates*		
Annual maturation rate, male	0.0271	^30^
Annual entry rate, male	0.0167	^30^
Annual mortality rate, male (background)	0.00486	^35^
*Annual mortality rate without ART*		
Acute	0.02	^36^
Asymptomatic (CD4 ≥ 500)	0.03	^36,37^
Symptomatic (200 < CD4 ≤ 500)	0.063	^36^
AIDS (CD4 ≤ 200)	0.22	^38,39^
*Annual mortality rate with ART*		
Acute with immediate start of ART	0.017	^36,37,40^
Asymptomatic (CD4 > 500) with ART	0.017	^36,37^
Symptomatic (200 < CD4 ≤ 500) with ART	0.05	^36^
AIDS (CD4≦200) with ART	0.075	^36^
**Biological**		
*Duration of HIV progression status converted to months*		
Acute to CD4 > 500	3	^41^
CD4 > 500 to 200 < CD4 ≤ 500	14.3	^41^
200 < CD4 ≤ 500 to CD4 ≤ 200	80.88	^41^
*Probability of HIV transmission per partnership per year*		
Acute (within 3 months)	0.17	Calculated ^a^^42^,
Asymptomatic (CD4 > 500)	0.024	Calculated ^a^^42^,
Symptomatic (200 < CD4≦500)	0.053	Calculated ^a^^42^,
AIDS	0.15	Calculated ^a^^42^,
*Reduction in infectivity (multiplicative) due to ART*		
Acute with immediate start of ART	0.10	^43^
Asymptomatic (CD4 > 500) on ART	0.05	^44^
Symptomatic (200 < CD4≦500) on ART	0.05	^44^
AIDS on ART	0.05	^44^
**Behavioral**		
*Annual number of partners*		
MSM, total	7.5	^5^
Low risk MSM	Calculated^b^	^5^
High risk MSM	14	^5^
Reduction in sexual behavior after HIV diagnosis	0.2	Assumed
Reduction in sexual behavior among AIDS patients	0.9	Assumed
**Condom use (% of sexual encounters)**		
Condom use before HIV diagnosis (%)	0.4	^5^
Condom use after HIV diagnosis (%)	0.6	^45^
Condom effectiveness	0.9	^45,46^
*HIV screening*		
Proportion of population tested in past 12 months, %	0.3	^2^
Rate of detection of HIV through passive case-finding	0.1	Assumed
Rate of detection of AIDS through passive case-finding	1	Assumed
**Policy and interventions**		
*Monthly entry rate to ART: base case*		
Acute	0.20	Calculated^c^
Asymptomatic (CD4 ≥ 500)	0.29	Calculated^c^
Symptomatic (200 < CD4 ≤ 500)	0.38	Calculated^c^
AIDS (CD4 ≤ 200)	0.43	Calculated^c^
*Monthly entry rate to ART: test and treat strategy*		
Acute	1	Assumed
Asymptomatic (CD4 ≥ 500)	1	Assumed
Symptomatic (200 < CD4 ≤ 500)	1	Assumed
AIDS (CD4 ≤ 200)	1	Assumed
**Costs (Japanese yen)**		
*Monthly HIV-related health care cost excluding ART*		
Acute	6,533	Calculated^d^
Asymptomatic (CD4 ≥ 500)	6,533	Calculated^d^
Symptomatic (200 < CD4 ≤ 500)	6,533	Calculated^d^
AIDS (CD4 ≤ 200)	1,016,936	Calculated^d^
*Monthly HIV diagnostic test cost*		
ELISA-	101	Calculated^e^
ELISA + plus WB	334	Calculated^e^
ELISA + plus WB—plus PCR	834	Calculated^e^
Behavioral counselling	235	Calculated^e^
*Monthly cost of PrEP*		
Drug cost	115,890	Calculated^f^
Initial clinic visit	5760	Calculated^f^
Subsequent clinic visits	2450	Calculated^f^

^a^Obtained from AIDS Clinical Center clinical cohort, converting average viral loads to infectiousness risk based on the model provided in Wilson et al.^42^.

^b^Partner numbers in low-risk and high–risk groups were calculated based on the proportion of people in each group to ensure an average number of partners of 5.5 in the whole population.

^c^Obtained using survival analysis of AIDS Clinical Center clinical cohort data.

^d^Obtained using average treatment costs from the AIDS Clinical Center clinical cohort.

^e^Based on the standard medical points schedule under the insurance system^47^, assuming one test per year and divided into monthly amounts.

^f^Obtained from AIDS Clinical Center clinical cohort data, assuming a cost of 3863 Japanese yen per tablet for TDF/FTC with no generic alternative.

Sensitivity analysis was conducted by randomly sampling some key parameters from a random distribution centred on the values described in Table 1. We ran 1000 models with 1000 randomly sampled sets of parameters and obtained prevalence estimates for all 1000 sample models. These prevalence estimates were calibrated against prevalence estimates for the years 2010–2016 using a calibration function based on the deviance statistic for Poisson distributions^21^. The 100 best-calibrated models were retained for the purpose of estimating uncertainty.

We solved the model numerically using a monthly time step and ran it forward in time to 2050. We obtained estimates of annual prevalence and incidence for a base-case model in which policies do not change from the current situation. Against this we tested seven alternative scenarios:

1. UNAIDS 90-90-90: A model in which Japan expands test and treat policies to match the UNAIDS 90-90-90 targets
2. PrEP 25%: PrEP provided to 25% of high-risk men for free, with 90% adherence and no risk compensation
3. PrEP 50%: PrEP provided to 50% of high-risk men for free, with 90% adherence and no risk compensation
4. PrEP 75%: PrEP provided to 75% of high-risk men for free, with 90% adherence and no risk compensation
5. UNAIDS 90-90-90 + Prep 25%: Japan expands test and treat policies to match the UNAIDS targets and offers PrEP to 25% of high-risk men with 90% adherence and no risk compensation
6. UNAIDS 90-90-90 + Prep 50%: Japan expands test and treat policies to match the UNAIDS targets and offers PrEP to 50% of high-risk men with 90% adherence and no risk compensation
7. UNAIDS 90-90-90 + Prep 75%: Japan expands test and treat policies to match the UNAIDS targets and offers PrEP to 75% of high-risk men with 90% adherence and no risk compensation

For all these scenarios we obtained estimates of the number of HIV infections averted relative to the base case, and the total cost of the strategy. Costs were estimated over the period to 2050, with a 3% discount rate and converted to international dollars and adjusted for inflation. We then calculated cost savings under the alternative policies. Because all strategies are cost saving, and because of the problems of estimating incremental cost effectiveness ratios (ICERs) when cost-effectiveness data cross more than one quadrant of the cost effectiveness plane, we did not perform cost-effectiveness analysis. All models were run in Matlab 2019a^22^.

## Results

Under the base case scenario, if current policies do not change and the HIV epidemic is allowed to continue among MSM without enhanced policy interventions, HIV prevalence would increase to 9.1% by 2050 (95% uncertainty interval (UI): 7.5–2.9%). All other policy interventions reduced long-term prevalence relative to the base case, and all other policies were cost-saving relative to the base case.

**Figure 2:**
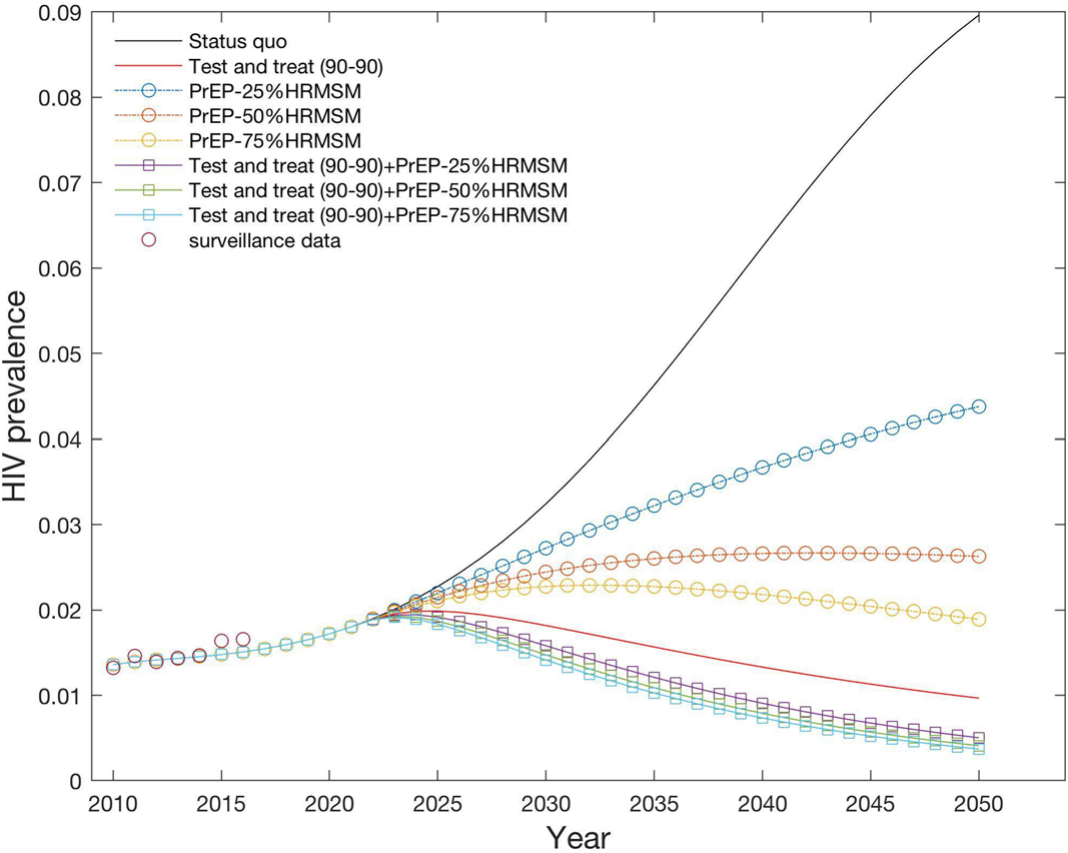
Prevalence until 2050 for 7 different scenarios.

Figure 2 shows the trends in prevalence for all scenarios. PrEP at 25–50% coverage reduces long-term prevalence but does not appear to have a major impact on the epidemic. Full achievement of 90-90-90 targets causes a rapid decline in HIV prevalence, and the additional epidemiological benefit of PrEP in combination with the UNAIDS strategy is limited.

Table 2 shows the prevalence in 2050, total number of HIV cases averted and annual cost saving (in international dollars) with 95% UI for all models. If no change is made to prevention policies there will be a total of 113,490 HIV infections by 2050 (95% UI: 91,020–180,980), representing a final prevalence of HIV among MSM of 9.0%. Achieving UNAIDS test and treat targets immediately would avert 84.9% of these future infections (95% UI: 81.1–85.6%), while even the least aggressive PrEP policy (25% coverage) would avert 44.2% of these infections (95% UI: 35.0–49.8%).

**Table 2:** Epidemiological impact and cost benefit of all policies until 2050.

	Total infections (1000 s)	HIV infections prevented (1000 s)	HIV infections prevented (%)	HIV prevalence at 2050, %	HIV incidence rate at 2050 (/100 PY)	Annual cost saving ($INT, millions)
The baseline case	113.5 (91.0, 180.98)	–	–	9.0 (7.5, 12.9)	0.86 (0.81, 1.07)	
Test and treat (90–90)	17.09 (13.08, 34.22)	96.40 (77.94, 146.76)	84.9% (81.1%, 85.6%)	1.0 (0.7, 2.0)	0.05 (0.03, 0.11)	155.22 (121.50, 266.60)
PrEP-25% HRMSM	63.34 (45.69, 117.72)	50.16 (45.34, 63.26)	44.2% (35.0%, 49.8%)	4.4 (3.2, 8.2)	0.40 (0.28, 0.71)	72.02 (59.86, 110.69)
PrEP-50% HRMSM	43.00 (30.59, 83.60)	70.49 (60.43, 97.38)	62.1% (53.8%, 66.4%)	2.6 (1.8, 5.3)	0.20 (0.12, 0.44)	102.52 (83.18, 166.95)
PrEP-75% HRMSM	33.64 (24.09, 65.62)	79.85 (66.94, 115.36)	70.4% (63.7%, 73.5%)	1.9 (1.3, 3.9)	0.12 (0.07, 0.28)	118.01 (94.68, 197.31)
Treat (90–90) + PrEP 25% HRMSM	9.95 (8.13, 18.12)	103.54 (82.89, 162.86)	91.1% (90.0%, 91.2%)	0.5 (0.4, 0.9)	0.01 (0.01, 0.02)	169.80 (132.49, 298.03)
Treat (90–90) + PrEP 50% HRMSM	7.96 (6.61, 14.05)	105.53 (84.41, 166.93)	92.7% (92.2%, 93.0%)	0.4 (0.4, 0.7)	0.01 (0.01, 0.01)	175.01 (136.52, 308.50)
Treat (90–90) + PrEP 75% HRMSM	6.98 (5.85, 12.12)	106.52 (85.17, 168.86)	93.6% (93.3%, 93.8%)	0.4 (0.3, 0.6)	0.01 (0.00, 0.01)	177.81 (138.71, 313.96)

Numbers in brackets show uncertainty intervals based on sensitivity analysis of key parameters in the model. All cost estimates are obtained after applying a 3% annual discount rate.

Because of the high cost of HIV treatment in Japan and the high incidence under the base case all tested strategies were cost saving, with savings ranging from 72.02 million $INT for PrEP (25% coverage) per year to 177.81 million $INT for UNAIDS 90-90-90 targets + PrEP (75% coverage). PrEP offers little additional benefit in either epidemiological outcome or cost benefit relative to just meeting UNAIDS targets, but by itself is still likely to offer large cost savings.

The combination strategies also showed the potential to reduce HIV incidence below 0.1 per 100 person years^23^, a common definition of elimination of HIV in deterministic models^19^. This suggests that the correct combination of these policies could be both cost-saving and offer a pathway to elimination.

## Discussion

This study implemented a deterministic, compartmental model describing the epidemiology of HIV in a population of Japanese MSM, and tested several options for enhancing the policy response to HIV in the Japanese context. The mathematical model presented in this study is the most comprehensive description of the HIV disease process in Japan that has been implemented to date. It incorporates an acute infection stage, allows for people living with HIV to be unidentified by testing, identified but not in treatment, or in treatment, and includes compartments for HIV negative individuals who know their status and are taking PrEP. The model approximately reflects Japanese treatment guidelines and the current reality of treatment procedures in Japan, and is informed by data from a clinical cohort of PLWH taken from Japan’s largest facility for the treatment and management of HIV.

Consistent with past research, this study found that without strengthened prevention efforts, if Japanese HIV policies remain unchanged, the HIV epidemic will continue to grow among Japanese MSM. In the base case where HIV counter-measures are not improved, prevalence of HIV was estimated to increase to 9.0% by 2050. This prevalence estimate is lower than an estimate from a previous mathematical model^12^, but is consistent with recent findings that the number of new infections may have stopped increasing^13^. However it still represents a significant increase on current prevalence and suggests that the epidemic is not contained, and more efforts are needed to control the spread of HIV among MSM.

This study tested the impact of scaling up testing and treatment practices in Japan to match the UNAIDS 90-90-90 targets, PrEP at three levels of coverage of high-risk MSM, and combinations of these strategies. Prevalence was projected forward until 2050 under a base case of no change in policy, and for all the alternative strategies and their combinations. We found that scaling up test-and-treat strategies to UNAIDS standards immediately would prevent 84.9% (UI: 81.1–85.6%) of new infections over the next 30 years, while in the absence of improved test and treat strategies PrEP programs covering at least 25% of high risk men would prevent at least 35% of new infections. Combinations of PrEP and test and treat would be even more effective, but the additional prevention benefits of these programs compared to enhanced test-and-treat strategies alone were limited. This is likely because once the 90-90-90 goal is met, the net benefit of PrEP is reduced, since many sexual contacts will experience the beneficial protection of testing and treatment even where one person in the contact is not taking PrEP. For example, under the 90-90-90 regime, PrEP can only benefit those who have sex with the 19% of PLWH who are not in treatment, with associated limited additional benefits.

Our economic analysis also found that all the strategies we tested were cost-saving, even in the case of low-coverage PrEP strategies that do not provide a large benefit in infections averted. Both enhanced testing and treatment and PrEP have been shown to be effective in preventing HIV transmission overseas^24^, to such an extent that in Japan any of these strategies, either in isolation or combination, could be expected to reduce the total cost of the HIV epidemic. We also found that the enhanced test and treat strategy would be likely to lead to effective elimination of new cases of HIV by 2050, and offers a policy pathway for achieving zero new infections in Japan within 30 years.

Our findings indicate a clear path forward for HIV policy in Japan. HIV prevention in Japan should shift to an immediate scale-up of testing, incorporating immediate entry into treatment into guidelines and supporting this process financially so that people who test positive for HIV are able to immediately enter into treatment and achieve rapid viral suppression. This strategy will require significantly increased rates of testing amongst MSM in order to ensure that all MSM at risk of HIV are tested regularly, and all newly-diagnosed MSM learn their serostatus rapidly. Because the scale up of test and treat strategies will take time and require additional infrastructure, as a short term strategy for HIV prevention the government should consider implementing a PrEP program for high-risk MSM, financed under the national insurance system or through approval of generic medicine at an affordable price, and enhanced testing and counselling for those most at risk. In the medium term, this enhanced HIV prevention strategy will require an increase in the capacity of the Japanese health care system to provide large numbers of HIV tests and expanded PrEP prescriptions in a short time.

One measure to increase test and treat capacity in Japan is to promote integration of collective sexual health care into community-based services. The HIV care facility network that covers Japan ensures nationwide access to ART, but sexual health services are limited and segmented into local clinics with different specialties such as genitourinary medicine, obstetrics and gynecology, or internal medicine. Since awareness of PrEP is very limited in such local settings even in areas with the most severe HIV epidemic such as Tokyo and Osaka^18^, increased focus on capacity-building for collective sexual health care including PrEP information for healthcare professionals is necessary. In addition, more financial support to enhance regular HIV tests at clinics is essential. Currently asymptomatic HIV tests are not covered by the national insurance system and require a significant copayment that is likely to undermine willingness for testing HIV both in physicians and in people at risk. In contrast to many European and Australian cities, Japan lacks comprehensive, government-funded sexual health services targeted at sexual minorities and providing anonymous, non-judgmental care that is free at the point of service. Consideration needs to be given to establishing and funding such services in major population centres. Stigma and discrimination remain important issues holding back a more comprehensive and effective HIV response in Japan, and in addition to the need to mainstream sexual health services, Japan urgently needs renewed action on this issue. Attention to the differential rights of MSM in Japan (including marriage rights), and clearer action to protect the legal rights of sexual minorities, are essential if the country is to continue to make progress on HIV and STI prevention. With the Olympics rapidly approaching, global attention will be focused on these issues, and comprehensive action is needed to ensure that Japan’s reputation for socioeconomic equality is extended to sexual minorities and other marginalized groups.

In the meantime, greater efforts are needed to lift barriers to prompt commencement of antiretroviral treatment (ART) in order to adopt the test and treat strategy in Japan, as well as enhanced infrastructure to monitor linkage from diagnosis to care. Current Japanese treatment guidelines recommend treatment for all PLWH, but the copayment for this therapy may be prohibitive for some MSM, especially young and high-risk MSM. A common process to ensure early entry to treatment is to obtain a disability certification card, which enables funding for ART with less copayment regardless of CD4 count. This process is administered under the Law for the Welfare of People with Physical Disabilities^25^, which describes the process for obtaining a disability card and associated reduced costs. However the process is onerous and usually takes 2 months, since it requires data on CD4 counts and viral loads at two time points at least 30 days apart, followed by consideration by a local municipal committee that issues the certification. As a result our model assumes an average of approximately 4 months from first diagnosis to undetectable viral load, which is unacceptably long given the scale of the epidemic in Japan. To improve this process, the *Law for the Welfare of People with Physical Disabilities* must be changed, which requires tremendous political effort and time. Additionally, there is no program that monitors success or failure of linkage to HIV care on a large scale. The government reported a recent increased uptake of on-line home testing services up to 85,000 per year but the rate of successful linkage to care among those tested and positive for HIV has not yet been identified^26^. Tools for connecting and reporting between clinics and testing service providers should be created to ensure optimal adaptation of online home testing services in the Japanese HIV program. Since the preventive strategies of test and treat and PrEP have already become the mainstream of HIV prevention policies globally, immediate action is needed to bring processes up to international standards.

This research has several weaknesses. It assumes immediate implementation of UNAIDS guidelines, and so likely overestimates the short-term effectiveness of the test and treat strategy. There is limited information about the final price of PrEP under a government-supported access program, but this likely underestimates the cost–benefit of this strategy, since we have used estimates of the current private-financed cost of the drug that would almost certainly be decreased by a government-run program. Our model also does not take into account the possible acceptability of PrEP in Japan, or include any variable measuring the time taken to implement a PrEP program and achieve high coverage or acceptability. There is limited evidence about acceptability of PrEP in Japan, but recent research indicates the possibility of some resistance to widespread uptake of this prevention strategy^27^. However, previous modeling has shown that PrEP can be effective even where adherence is sub-optimal^19^, and even low levels of coverage of PrEP were still cost saving in this model, so it is unlikely that low acceptability will render PrEP ineffective in Japan. The model is dependent on the quality of parameters input to the model. This challenge is particularly important for information on condom use and sex partner numbers, two crucial drivers of epidemic behavior in the model that are not well understood in Japan. We have attempted to address this limitation through sensitivity analysis, which has led to wide uncertainty ranges around some of our final estimates. This model also is quite simplistic compared to other models that have been developed recently which take into account network effects and concurrency of partnerships^28,29^. Although the compartmental modeling structure used in this model allows for important basic policy analysis and remains useful for comparing interventions, it does not properly take account of the rich and diverse nature of human sexual relations, which are better modeled using more modern tools that incorporate partner networks, timing of partnerships, and differential risk patterns within sexual networks. Unfortunately the data which is needed for the development of such models is simply not available in Japan, and the lack of information on details of sexual activity in Japan holds back more sophisticated understanding of how social behavior, stigma, socio-sexual mores and health policy interact in the context of the HIV epidemic in Japan. To better understand how policy can affect sexual health and HIV risk, Japan urgently needs better information about sexual risk behavior and partner numbers in MSM, and especially in low-risk MSM. HIV prevention cannot rely on biomedical interventions alone, and while programs are scaled up in Japan and treatment as prevention remains slow to access, condom use and other changes in sexual risk behavior remain essential to the HIV response. Better understanding of condom use behaviors, and effective strategies to encourage them, are therefore urgently needed. Finally, Japan’s population is aging rapidly and the demographics of the MSM population are not well understood. The possible rapid maturation and lack of information about sexual activity in elderly MSM means long-term projections of HIV prevalence may be affected by slow changes in demographic and risk profiles of the population we are studying.

## Conclusion

This study provides strong evidence in support of the urgent need to scale up testing and treatment access among MSM in Japan. By taking rapid action to meet the goals of the UNAIDS 90-90-90 program; changing treatment guidelines in line with global best practice; and licensing PrEP for use among high-risk MSM, Japan can not only reduce the long-term burden of HIV/AIDS in Japan, but can effectively eliminate the disease among MSM and reduce the cost it poses. Our study gives clear evidence that the time has come for Japan to take the next step in HIV prevention, and join other developed nations in moving to end this epidemic.
